# Editorial: Genomic and evolutionary analysis of microsporidian parasites

**DOI:** 10.3389/fmicb.2023.1248661

**Published:** 2023-07-07

**Authors:** Qiang Huang, Hamed Mirjalali, Zeyang Zhou

**Affiliations:** ^1^Honeybee Research Institute, Jiangxi Agricultural University, Nanchang, China; ^2^Foodborne and Waterborne Diseases Research Center, Research Institute for Gastroenterology and Liver Diseases, Shahid Beheshti University of Medical Sciences, Tehran, Iran; ^3^State Key Laboratory of Resource Insects, Southwest University, Chongqing, China; ^4^Key Laboratory of Conservation and Utilization of Pollinator Insect of the Upper Reaches of the Yangtze River (Co-construction by Ministry and Province), Ministry of Agriculture and Rural Affairs, Chongqing Normal University, Chongqing, China

**Keywords:** microsporidia, intracellular parasite, spore forming, regulatory RNA, recombination, competition

Microsporidia (phylum Microspora) is a large, diverse group of obligate intracellular, spore-forming parasites related to fungi that can infect animals, including humans (Weiss and Becnel, 2014; Choi and Kim, 2017). Besides clinical importance, microsporidia infection particularly damages the apicultural, fishery, and silk industries. The genomic size of microsporidia varies from 2.3 to 53.1 Mb, which reduction and expansion were driven by multiple mechanisms, such as loss of genes and non-coding regions, gene and segmental duplications, horizontal gene transfers, and so on (Williams et al., 2022). The unique structure of microsporidia, like infecting apparatus polar tube, mitochondrial remnants termed mitosome, prokaryotic-like ribosome and one nucleus or diplokaryon makes them an enigmatic organism. The empirical data on ploidy and recombination remain deficient. The lack of genetic engineering tools and host cell culture further hinders the functional study of the taxa.

The Research Topic on “*Genomic and evolutionary analysis of microsporidian parasites*” aims to enhance the understanding of functional genomics, evolution, and host-parasite interaction of the microsporidian parasites. The collected 6 original researches are summarized below.

The first article on this topic assembled the genome of *Nosema ceranae*, a honey bee gut parasite, using long reads (Huang et al.). The compacted 8.8 Mbp genome consists 2,280 protein-coding genes. Various transporters are annotated, supporting the resource transfer from the host. *N. ceranae* maintained a complete RNAi pathway, which is absent in most microsporidia (Huang, 2018). The identified ATP transporter, polar tube proteins, sporoplasm surface protein, and *dicer* were essential for the proliferation (Paldi et al., 2010; Rodriguez-Garcia et al., 2018; Huang et al., 2019).

As an intracellular parasite, it is not surprising that *N. ceranae* regulates host gene expression. *N. ceranae* regulates the honey bee through signaling peptides or regulatory RNAs (Dolgikh et al., 2019; Shao et al., 2021). Fan et al. find that *N. ceranae* targets crucial cellular and humoral immune pathways of the honey bees through miRNA. Additionally, *N. ceranae* derived miRNA modulates the honey bee metabolism, facilitating proliferation and suggesting a cross-kingdom regulation.

*Nosema muscidifuracis* infects the parasitoid wasp *Muscidifurax zaraptor* and *Muscidifurax raptor*. Xiong et al. assemble a high continuity genome with only 28 contigs and develop a tool to diagnose the infection. Heavy infection occurs in the ovary, suggesting maternal-offspring transmission.

High genetic diversity is found in the parasite *Pseudokabatana alburnus*, infecting the fish *Culter alburnus* (Weng et al.). The haplotype analysis suggests that *P. alburnus* have intergenomic variation and recombination. The phylogenetic analysis suggests a minor variation among geographically distinct populations. High genetic diversity is also found in *N. ceranae*. Wei et al. revealed allelic oscillation through transgenerational analysis. The dynamic of the allele frequency supports the red queen hypothesis during *N. ceranae* and honey bee co-evolution, where the essential genes regulating ATP binding and apoptosis are constantly under selection.

It is common that the hosts share habitats. Orlansky and Ben-Ami find that the parasite regulates host competition. *Daphnia magna* competitively excludes *Daphnia similis* when they coexist in a habitat. Once the parasite *Hamiltosporidium tvaerminnensis* engages, the competitive advantage of *D. magna* dramatically decreases. The results suggest that *H. tvaerminnensis* shapes the host community, allowing the coexistence of the hosts, which provides broader insight into the ecological importance of the parasite.

This topic covers comparative genomics, genetic diversity, and host regulation of microsporidian parasites. We hope this topic provides a state-of-the-art reference to the emerging field of microsporidian genetics and ecology.

## Author contributions

All authors listed have made a substantial, direct, and intellectual contribution to the work and approved it for publication.
